# Minocycline Enhances the Effectiveness of Nociceptin/Orphanin FQ during Neuropathic Pain

**DOI:** 10.1155/2014/762930

**Published:** 2014-09-03

**Authors:** Katarzyna Popiolek-Barczyk, Ewelina Rojewska, Agnieszka M. Jurga, Wioletta Makuch, Ferenz Zador, Anna Borsodi, Anna Piotrowska, Barbara Przewlocka, Joanna Mika

**Affiliations:** ^1^Department of Pain Pharmacology, Institute of Pharmacology, Polish Academy of Sciences, 12 Smetna Street, 31-343 Cracow, Poland; ^2^Institute of Biochemistry, Biological Research Center, Hungarian Academy of Sciences, Temesvári krt 62 Street, Szeged 6726, Hungary

## Abstract

Nociceptin/orphanin FQ (N/OFQ) antinociception, which is mediated selectively by the N/OFQ peptide receptor (NOP), was demonstrated in pain models. In this study, we determine the role of activated microglia on the analgesic effects of N/OFQ in a rat model of neuropathic pain induced by chronic constriction injury (CCI) to the sciatic nerve. Repeated 7-day administration of minocycline (30 mg/kg i.p.), a drug that affects microglial activation, significantly reduced pain in CCI-exposed rats and it potentiates the analgesic effects of administered N/OFQ (2.5–5 *μ*g i.t.). Minocycline also downregulates the nerve injury-induced upregulation of NOP protein in the dorsal lumbar spinal cord. Our *in vitro* study showed that minocycline reduced *NOP* mRNA, but not protein, level in rat primary microglial cell cultures. In [^35^S]GTP*γ*S binding assays we have shown that minocycline increases the spinal N/OFQ-stimulated NOP signaling. We suggest that the modulation of the N/OFQ system by minocycline is due to the potentiation of its neuronal antinociceptive activity and weakening of the microglial cell activation. This effect is beneficial for pain relief, and these results suggest new targets for the development of drugs that are effective against neuropathic pain.

## 1. Introduction

Neuropathic pain is a common consequence of nervous tissue damage. The mechanisms underlying neuropathy still remain unclear, and the currently available drugs are frequently ineffective, making treatment a major clinical challenge [1, 2]. Nociceptin/orphanin FQ (N/OFQ) acts through the N/OFQ peptide receptor (NOP) [3] and can change responsiveness to painful stimuli in several models of pain [4–6]. Pro- and antinociceptive effects of N/OFQ have been reported in a variety of animal models depending on the route of administration. Intracerebroventricular (i.c.v.) administration was found to display hyperalgesic effects [3, 7, 8], which were mediated by NOP, as these effects are not present in NOP-knockout mice [9]. In contrast, N/OFQ administered intrathecally (i.t.) has been generally found to produce antinociceptive responses [4, 5, 10–16].

The role of N/OFQ in the development of neuropathic pain has been extensively studied. In recent studies, it has been found that the N/OFQ system influences glial cell functions [17–21] and now it is considered if neuroimmune interaction may be one of the mechanisms of its antinociceptive properties. We have shown that microglia are the glial cell type to be activated in response to peripheral nerve injury and that this activation is in parallel with changes in neuropeptide systems involved in nociceptive transmission (proenkephalin, prodynorphin, and pronociceptin) [17]. Biochemical studies revealed the presence of N/OFQ and its receptor in the CNS and peripheral tissues, particularly in regions associated with nociceptive pathways [22–28]. The NOP was demonstrated to be expressed not only on neurons but also on astrocytes and microglia [20, 21], which further suggest the involvement of these cells in the modulation of N/OFQ system. Fu et al. have [20] shown that spinal cord astrocyte activation and* in vitro* cytokine production by those glial cells are attenuated by N/OFQ through the astrocytic NOP. It was shown that LPS-induced IL-1*β* gene expression was reduced by N/OFQ in cultured primary microglia, but it was enhanced in neuronal cultures [21]. The regulatory effects of N/OFQ on glia-derived cytokines suggest that the action of the N/OFQ system is dependent on glial cell activation in the CNS.

The aim of the present study was to determine the role of activated microglia in the analgesic effects of N/OFQ in a rat model of neuropathic pain (achieved by chronic constriction injury to the sciatic nerve, CCI). In our studies, we used minocycline, a well-characterized drug for inhibiting microglial activation [29–32], viability, and migration [33, 34]. Minocycline (30 mg/kg) was intraperitoneally (i.p.) administered preemptively 16 h and 1 h before CCI and then twice daily for 7 days. On day 7 after injury, vehicle- or minocycline-treated CCI-exposed rats received intrathecally (i.t.) N/OFQ, and we examined the analgesic effects using von Frey and cold plate tests. In our biochemical studies, we analyzed molecular changes in mRNA and protein levels of the NOP in the dorsal horn of the lumbar spinal cord at day 7 after injury in vehicle- or minocycline-treated CCI-exposed rats using qRT-PCR and Western blot analysis, respectively. Additionally, using primary cultures of rat microglia, we investigated the effects of minocycline on mRNA and protein levels of NOP. We also investigated the effects of minocycline on NOP signaling in the spinal cord of the vehicle- or minocycline-treated CCI-exposed rats using a functional [^35^S]GTP*γ*S binding assay.

## 2. Methods

### 2.1. Animals

Male Wistar rats (200–350 g) were housed in cages that were lined with sawdust under a standard 12/12 h light/dark cycle (lights on at 08:00 h) with food and water available* ad lib*. Care was taken to reduce the number of animals used. All experiments were approved by the local bioethics committee (Cracow, Poland) and were performed according to the recommendations of IASP [35], ARRIVE guidelines [36], and the NIH Guide for the Care and Use of Laboratory Animals.

### 2.2. Surgical Preparations

Chronic constriction injury (CCI) was produced in rats according to Bennett and Xie [37], by tying four ligatures around the sciatic nerve under sodium pentobarbital anesthesia (60 mg/kg; i.p.). The* biceps femoris* and the* gluteus superficialis* were separated, and the right sciatic nerve was exposed. The ligatures (4/0 silk) were tied loosely around the nerve distal to the sciatic notch with 1-mm spacing until they elicited a brief twitch in the respective hind limb. After the surgery, all rats developed long-lasting neuropathic pain symptoms such as allodynia and hyperalgesia. Because we have shown in earlier studies that there are no differences between the nociceptive responses of naïve and sham animals [38], we used naïve animals for the behavioral experiments in the present study.

### 2.3. Intrathecal (i.t.) Injection

Rats were prepared for intrathecal (i.t.) injection by implanting catheters according to the method of Yaksh and Rudy [39] under pentobarbital (60 mg/kg i.p.) anesthesia. The intrathecal catheter consisted of polyethylene tubing that was 12 cm long (PE 10, Intramedic; Clay Adams, Parsippany, NJ) with an outside diameter of 0.4 mm and a dead space of 10 *μ*L that had been sterilized by immersion in 70% (v/v) ethanol and been fully flushed with sterile water before insertion. Rats were placed on a stereotaxic table (David Kopf Instruments, Tujunga, CA), and an incision was made in the atlantooccipital membrane. The catheter (7.8 cm of its length) was carefully introduced into the subarachnoid space at the rostral level of the spinal cord lumbar enlargement (L4-L5). After the implantation, the first injection of 10 *μ*L of water was performed slowly, and the catheter was tightened. After catheter implantation, the rats were monitored for physical impairments. Those showing motor deficits (ca 5%) were excluded from further study. Animals were allowed a minimum of 1 week to recover after the surgery before the experiment began. Water for injection or respective drugs were delivered slowly (1-2 min) in a volume of 5 *μ*L through the i.t. catheter and were followed by 10 *μ*L of water, which flushed the catheter.

### 2.4. Behavioral Tests

#### 2.4.1. Tactile Allodynia (Von Frey Test)

Allodynia was measured in rats subjected to CCI by the use of an automatic von Frey apparatus (Dynamic Plantar Aesthesiometer cat. no. 37400, Ugo Basile, Italy). Rats were placed in plastic cages with a wire net floor 5 min before the experiment. The von Frey filament (up to 26 g) was applied to the midplantar surface of the hind foot, and measurements were taken automatically [40].

#### 2.4.2. Hyperalgesia (Cold Plate Test)

Hyperalgesia was assessed using the cold plate test (Cold/Hot Plate Analgesia Meter no. 05044 Columbus Instruments, USA) as has been described previously [40, 41]. The temperature of the cold plate was maintained at 5°C, and the cut-off latency was 30 s. The rats were placed on the cold plate, and the time until lifting of the hind foot was recorded. The injured foot was the first to react in every case.

### 2.5. Drug Administration

The chemicals used in this study and their sources were as follows: N/OFQ (cat. no. 0910, TOCRIS, UK), and minocycline hydrochloride (cat. no. M9511, Sigma-Aldrich, USA). Minocycline (30 mg/kg; i.p.) was dissolved in sterile water and preemptively administered intraperitoneally 16 h and 1 h before CCI and then twice daily for 7 days. This method of minocycline administration was used throughout the work and is referred to in the text as “repeated administration”. The control groups received a vehicle (water for injection) according to the same schedule. One hour after the last morning of minocycline or vehicle administration on day 7 after CCI, N/OFQ (2.5 and 5 *μ*g/5 *μ*L) or a vehicle was i.t. injected. After vehicle or N/OFQ administration the von Frey test (20 and 40 min later) and cold plate tests (30 and 50 min later) were performed.

### 2.6. Microglial Cell Cultures and Treatments

Primary cultures of microglial cells were prepared from 1-day-old Wistar rat pups as previously described [42]. Briefly, cells were isolated from the rats' cerebral cortices and were plated at a density of 3 × 10^5^ cells/cm^2^ in a culture medium that consisted of DMEM/Glutamax/high glucose (Gibco, USA) supplemented with heat-inactivated 10% fetal bovine serum (Gibco, USA), 100 U/mL penicillin, and 0.1 mg/mL streptomycin (Gibco, USA) on poly-L-lysine coated 75 cm^2^ culture flasks maintained at 37°C and 5% CO_2_. The culture medium was changed after 4 days. The loosely adherent microglial cells were recovered after 9 days by mild shaking and centrifugation. Microglial cells were suspended in a culture medium and plated at a final density of 2 × 10^5^ cells onto 24-well plates and 1.2 × 10^6^ cells onto 6-well plates. Adherent cells were incubated for 48 h in a culture medium before being used for the analyses. Cell specificity was determined in cultures of primary microglia by Western blot assay using an antibody against OX-42 (a microglial marker) and qRT-PCR using primers for* C1q* (a microglial marker) and* GFAP* (an astrocyte marker). The homogeneity of microglial population was kept on high level (more than 95% positive for OX-42 and C1q), and our homogeneity was similar to those published by Mika et al. [41]. Primary microglial cell cultures were treated with minocycline (10 *μ*M) or vehicle (water) for 6 h for mRNA analysis and for 24 h for protein analysis.

### 2.7. qRT-PCR Analysis of Gene Expression

Ipsilateral dorsal rat spinal cords (L4–L6) were collected 7 days after injury, 4 h after last morning minocycline treatment. Total RNA was extracted according to the method described by Chomczynski and Sacchi [43] using TRIzol reagent (Invitrogen) as previously described [6]. RNA concentration was measured using a NanoDrop ND-1000 Spectrometer (NanoDrop Technologies). Reverse transcription was performed on 500 ng (from cell cultures) or 1000 ng (from tissue) of total RNA using Omniscript reverse transcriptase (Qiagen Inc.) at 37°C for 60 min. cDNA was diluted 1 : 10 with H_2_O. qRT-PCR was performed using Assay-On-Demand TaqMan probes according to the manufacturer's protocol (Applied Biosystems) and run on a Real-Time PCR iCycler (BioRad, Hercules, CA, USA). Rn01527838_g1 (*Hprt*) and Rn00440563_m1 (*Orl1*) were used as TaqMan primers and probes. The expression of HPRT (a housekeeping gene) was quantified to control group for variation in cDNA amounts. Cycle threshold values were calculated automatically by iCycler IQ 3.0 software with default parameters. Abundance of RNA was calculated as 2^−(threshold  cycle⁡)^.

### 2.8. Western Blot Analysis

Ipsilateral dorsal rat spinal cords (L4–L6) were collected for protein analyses at day 7 after injury, 6 h after the last morning minocycline treatment. Cell and tissue lysates were collected in RIPA buffer with a protease inhibitor cocktail and cleared by centrifugation (14000 ×g for 30 min, 4°C). Samples containing 15 *μ*g (cells lysates) and 20 *μ*g (tissue lysates) of protein were heated in a loading buffer (50 mM Tris-HCl, 2% SDS, 2% *β*-mercaptoethanol, 4% glycerol, and 0.1% bromophenol blue) for 8 min at 98°C and resolved on 10–20% Criterion TGX precast polyacrylamide gels. Following gel electrophoresis, the proteins were transferred to Immune-Blot PVDF membranes (Bio-Rad) with semidry transfer (30 min, 25 V). The membranes were blocked for 1 h using 5% nonfat dry milk (Bio-Rad) in Tris-buffered saline with 0.1% Tween 20 (TBST), washed in TBST, incubated overnight at 4°C with primary antibodies (rabbit polyclonal anti-OPRL1, 1 : 600; rabbit polyclonal anti-IBA1, 1 : 500, ProteinTech), and incubated for 1 h at RT with a secondary goat polyclonal antibody that had been conjugated to horseradish peroxidase (goat anti-rabbit IgG, 1 : 5000, BioRad). Both primary and secondary antibodies were diluted in solutions from SignalBoost Immunoreaction Enhancer Kit (Merck Millipore). Membranes were washed 2 × 2 min and 3 × 5 min with TBST. Immunocomplexes were detected using a Immun-Star HRP Chemiluminescent Substrate Kit (BioRad) and visualized using a Fujifilm LAS-4000 FluorImager system. The blots were stripped using Restore Western Blot Stripping Buffer (ThermoScientific) for 15 min at RT, washed in TBST, and reprobed with a mouse antibody against GAPDH (1 : 5000, Millipore) as a loading control. The relative levels of immunoreactivity were quantified using Fujifilm Multi Gauge software.

### 2.9. Immunocytochemical Analysis

We used commercially available specific anti-NOP antibodies. Cells were fixed for 20 minutes in 4% paraformaldehyde in a 0.1 M phosphate buffer (pH 7.4) and incubated with primary antibodies (rabbit anti-ORL-1, 1 : 500, ProteinTech) for 2 days at 4°C. After three washes in phosphate buffered saline (PBS), immunofluorescence was revealed by incubation for 2 h in the fluorochrome-conjugated secondary antibody, Alexa Fluor555 donkey, antirabbit diluted 1 : 500 in 5% NDS. Sections were then washed with PB and coverslipped with an Aquatex mounting medium (Merck, Darmstadt, Germany). Sections without primary antibodies were used as negative controls.

### 2.10. Functional [^35^S]GTP*γ*S Binding Assay

Ipsilateral dorsal rat spinal cords (L4–L6) were collected 7 days after injury, 6 h after the last morning minocycline treatment and were prepared for the assay as previously described [44] with modifications. The membrane fractions of rat spinal cords were diluted in TEM buffer (50 mM Tris-HCl, 1 mM EGTA, and 5 mM MgCl_2_; pH 7.4) to achieve the appropriate protein content for the assays (~10 *μ*g of protein/sample).

The [^35^S]GTP*γ*S assays were prepared according to Sim et al. [45] and Traynor and Nahorski [46] with slight modifications. The membrane fractions were incubated at 30°C for 60 min in Tris-EGTA buffer (composed of 50 mM Tris-HCl, 1 mM EGTA, 3 mM MgCl_2_, and 100 mM NaCl; pH 7.4). The buffer also contained 20 MBq/0.05 cm^3^ [^35^S]GTP*γ*S (0.05 nM) and increasing concentrations (10^−10^–10^−5 ^M) of N/OFQ 1–17 in the presence of excess GDP (30 *μ*M) in a final volume of 1 mL. Total binding (T) was measured in the absence of N/OFQ 1–17, while nonspecific binding (NS) was determined in the presence of 10 *μ*M unlabeled GTP*γ*S and subtracted from the total binding. The difference (T-NS) represents basal activity. Bound and free [^35^S]GTP*γ*S were separated by vacuum filtration through Whatman GF/B filters with Brandel M24R cell harvester. Filters were washed three times with 5 mL ice-cold buffer (pH 7.4), and the radioactivity of the filters was detected in UltimaGold MV aqueous scintillation cocktail with Packard Tricarb 2300TR liquid scintillation counter. [^35^S]GTP*γ*S binding experiments were performed in triplicates and repeated at least three times.

### 2.11. Data Analysis

The behavioral data are presented as the mean ± SEM of 8–16 rats per group. The results of the experiments were statistically evaluated using one-way analysis of variance (ANOVA). All of the differences between the treatment groups were further analyzed with Bonferroni's* post hoc* tests. Significant differences in comparisons with vehicle-treated CCI-exposed rats are indicated by *P(<0.05), **P(<0.01), and ***P(<0.001). Significant differences between vehicle-treated CCI-exposed rats that had received a single dose of N/OFQ and minocycline-treated CCI-exposed rats that had received a single dose of N/OFQ are indicated by ^#^ *P*(<0.05) and ^###^ *P*(<0.001).

The qRT-PCR analyses from the tissue were performed in three groups: naïve, vehicle-treated CCI-exposed, and minocycline-treated CCI-exposed rats. The results from 6–8 animals are presented as fold changes compared with the naïve rats in the ipsilateral dorsal lumbar spinal cord. The results from 4 cell cultures are presented as fold changes compared with vehicle-treated cells. The qRT-PCR data are presented as the mean ± SEM and represent the normalized averages that were derived from the threshold qRT-PCR cycles from four to eight samples for each group. Intergroup differences were analyzed using ANOVAs followed by Bonferroni's multiple comparison tests. In the cell cultures analysis, the intergroup differences were analyzed by *t*-test; significant differences resulting from comparisons with nonstimulated cells are indicated by *P(<0.05).

The protein analyses were performed using Western blots. The analyses from the tissue were performed in three groups: naïve, vehicle-treated CCI-exposed, and minocycline-treated CCI-exposed rats. The results are presented as fold changes compared to the naïve rats in the ipsilateral dorsal lumbar spinal cord. The results from cell cultures are presented as fold changes compared with vehicle-treated cells. The data are presented as the mean ± SEM and represent the normalized averages derived from analyses of four to five samples for each group (for tissue analysis) and four cell cultures performed with the Multi Gauge analysis program. Intergroup differences were analyzed using ANOVA followed by Bonferroni's multiple comparison tests. **P(<0.01) indicates significant differences compared to naïve rats. ^#^ *P*(<0.05) indicates significant differences compared to the CCI-treated group. In cell cultures analysis intergroup differences were analyzed with *t*-test.

In the [^35^S]GTP*γ*S binding assays, the specifically bound [^35^S]GTP*γ*S were presented as percentages as the function of the applied concentrations of N/OFQ 1–17 in logarithmic scale. Basal activity was settled as 100%; experimental data are presented as the mean ± S.E.M. The data were fitted with GraphPad Prism 5.0 (GraphPad Prism Software Inc., San Diego, CA) curve fitting program to determine the maximal stimulation or efficacy (*E*_max⁡_) of the receptor mediated G-protein and the potency (EC_50_) of the stimulator ligand. Statistical analysis was performed using one-way ANOVA with Bonferroni's multiple comparison* post hoc* test to determine the significance level. Significance was accepted at the *P(<0.05) level.

## 3. Results

### 3.1. Repeated Minocycline Administration Diminished the Development of Neuropathic Pain and Enhanced the Effectiveness of Nociceptin/Orphanin FQ

**Figure 1:**
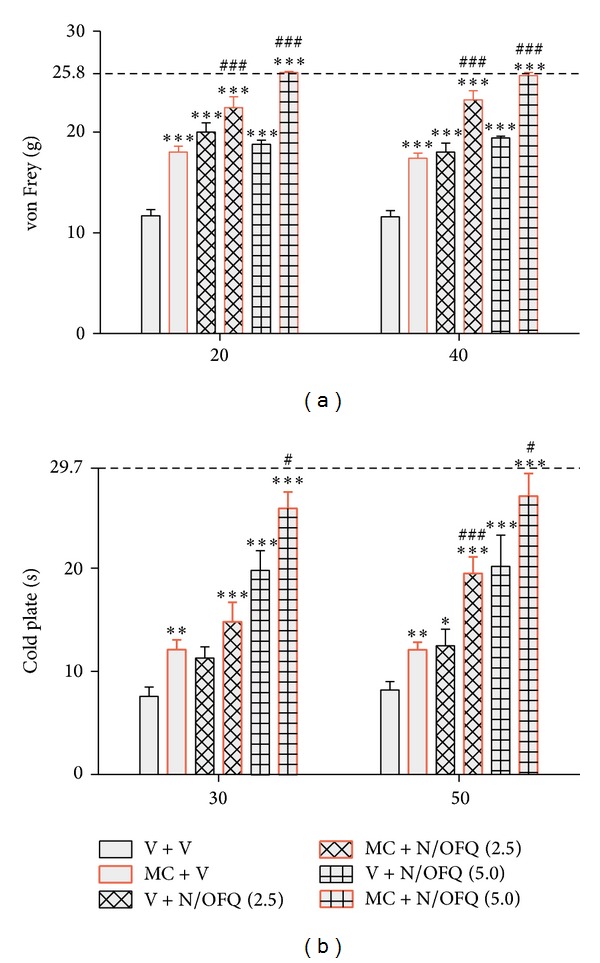
Repeated minocycline administration diminished the development of neuropathic pain and enhanced the effectiveness of N/OFQ. The response to N/OFQ was measured 20 and 40 minutes after administration by the von Frey test (a) and 30 and 50 minutes after administration by the cold plate test (b). Minocycline (MC; 30 mg/kg; i.p.) was administered intraperitoneally preemptively 16 h and 1 h before CCI and then repeatedly twice daily for 7 days. Vehicle-treated and minocycline-treated rats received intrathecal N/OFQ (2.5; 5 *μ*g/5 *μ*L) one hour after the last morning administration on day 7 after CCI. The data are presented as the mean response ± SEM. (8–16 rats per group). The results of the experiments were statistically evaluated using one-way analyses of variance (ANOVA). The differences between the treatment groups throughout the study were further analyzed with Bonferroni's* post hoc* tests. *P < 0.05, **P < 0.01, and ***P < 0.001 indicate significant differences compared with vehicle-treated CCI-exposed rats; ^#^ *P* < 0.05 and ^###^ *P* < 0.001 indicate significant differences between vehicle-treated CCI-exposed rats that received a single dose of N/OFQ and minocycline-treated CCI-exposed rats that received a single dose of N/OFQ. The dotted line is a value for naïve animals (von Frey test 25.8 g; cold plate test 29.7** **s).

In the behavioral tests, all vehicle-treated CCI-exposed rats exhibited neuropathic pain symptoms. Seven days after injury, rats exhibited strong allodynia as measured by the von Frey test (11.7 g ± 0.6 compared with 25.8 g ± 0.2 for naïve rats) (Figure 1(a)) and potent hyperalgesia as measured by the cold plate test (7.6 s ± 0.9 compared with 29.7 s ± 0.3 for naïve rats) (Figure 1(b)). Minocycline (30 mg/kg; i.p.) administered repeatedly was effective in reducing mechanical allodynia as measured by the von Frey test (vehicle-treated 11.7 g ± 0.6 versus minocycline-treated 18.0 g ± 0.6) (Figure 1(a)) and also in reducing cold hyperalgesia as measured by the cold plate test (vehicle-treated 7.6 s ± 0.9 versus 12.1 s ± 0.9 minocycline-treated) (Figure 1(b)).

N/OFQ (2.5 and 5 *μ*g; i.t.) was injected at day 7 at one hour after the last morning dose of minocycline (30 mg/kg; i.p.) or vehicle. The effect of N/OFQ (2.5 *μ*g; 5 *μ*g/*μ*L; i.t.) in minocycline-treated rats as compared with vehicle-treated ones was significantly increased in the von Frey test 20 and 40 minutes after injection (Figure 1(a)). The antihyperalgesic effect of the lower dose of N/OFQ (2.5 *μ*g/*μ*L; i.t.) in minocycline-treated rats compared with vehicle-treated rats was significantly upregulated only after 50 minutes, while at a higher dose (5 *μ*g/*μ*L; i.t.) the antihyperalgesic effect was potentiated at both times (Figure 1(b)).

### 3.2. Repeated Minocycline Administration Influenced the Nociceptin/Orphanin FQ System Parallel to Microglia Regulation in the Spinal Cord Level under Neuropathic Pain

**Figure 2:**
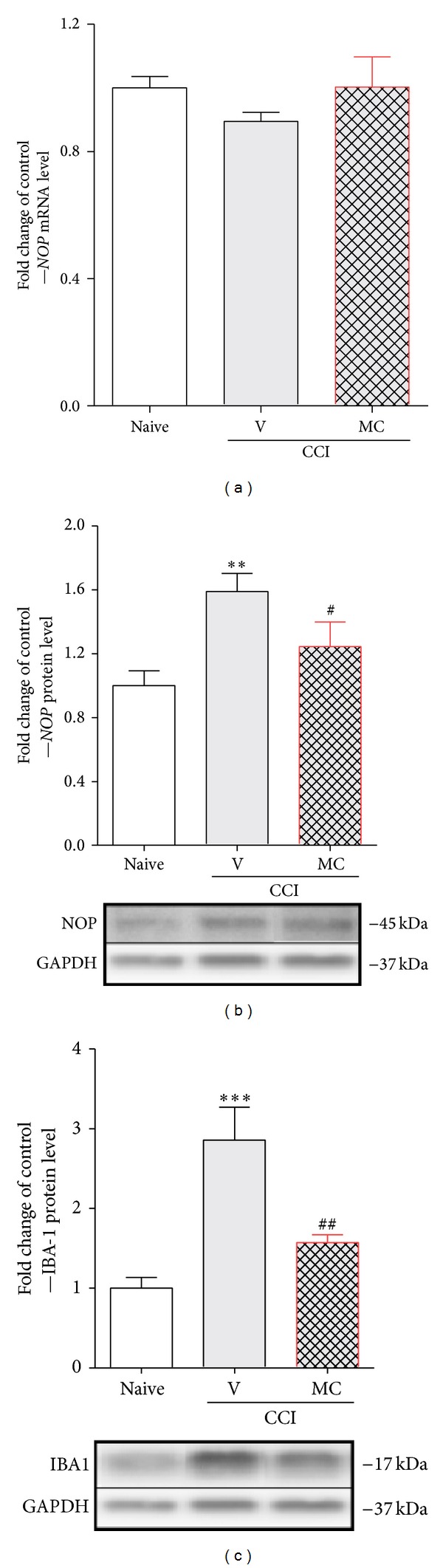
Repeated minocycline administration influenced the N/OFQ system parallel to microglia regulation in the spinal cord level under the neuropathic pain. Seven days after CCI in the ipsilateral dorsal spinal cord, minocycline-treatment diminished the level of NOP (b) and IBA-1 (c) proteins levels that were upregulated by nerve injury. The* NOP* mRNA level was unchanged by nerve injury and minocycline treatment (a). The qRT-PCR and Western blot data are presented as the mean ± SEM and represent the normalized averages derived from analyses of 4–8 samples for each group. Intergroup differences were analyzed using ANOVA followed by Bonferroni's multiple comparison test. **P < 0.01 and ***P < 0.001 indicate significant differences compared with naïve rats. ^#^ *P* < 0.05 and ^##^ *P* < 0.01 indicate significant differences comp ared with the CCI-treated group. V: vehicle, MC: minocycline.

Seven days after CCI in the ipsilateral lumbar dorsal spinal cord an increase of 60% of NOP protein was observed compared with naive animals (Figure 2(b)). Repeated administration of minocycline reduced the upregulation of NOP protein level (by 24%) in comparison with naive rats (Figure 2(b)). No changes in* NOP* mRNA were seen in the vehicle- or the minocycline-treated rats compared with naive animals (Figure 2(a)). Parallel to NOP protein regulation, we observed the upregulation of protein by 185% for IBA-1 (a microglial marker) in the ipsilateral lumbar dorsal spinal cord in the vehicle-treated rats (Figure 2(c)). Repeated administration of minocycline diminished the level of microglial activation marker to 57% in comparison with naive rats (Figure 2(c)).

### 3.3. Repeated Minocycline Administration Influenced Nociceptin/Orphanin FQ Peptide Receptor Signaling under Neuropathic Pain

**Figure 3:**
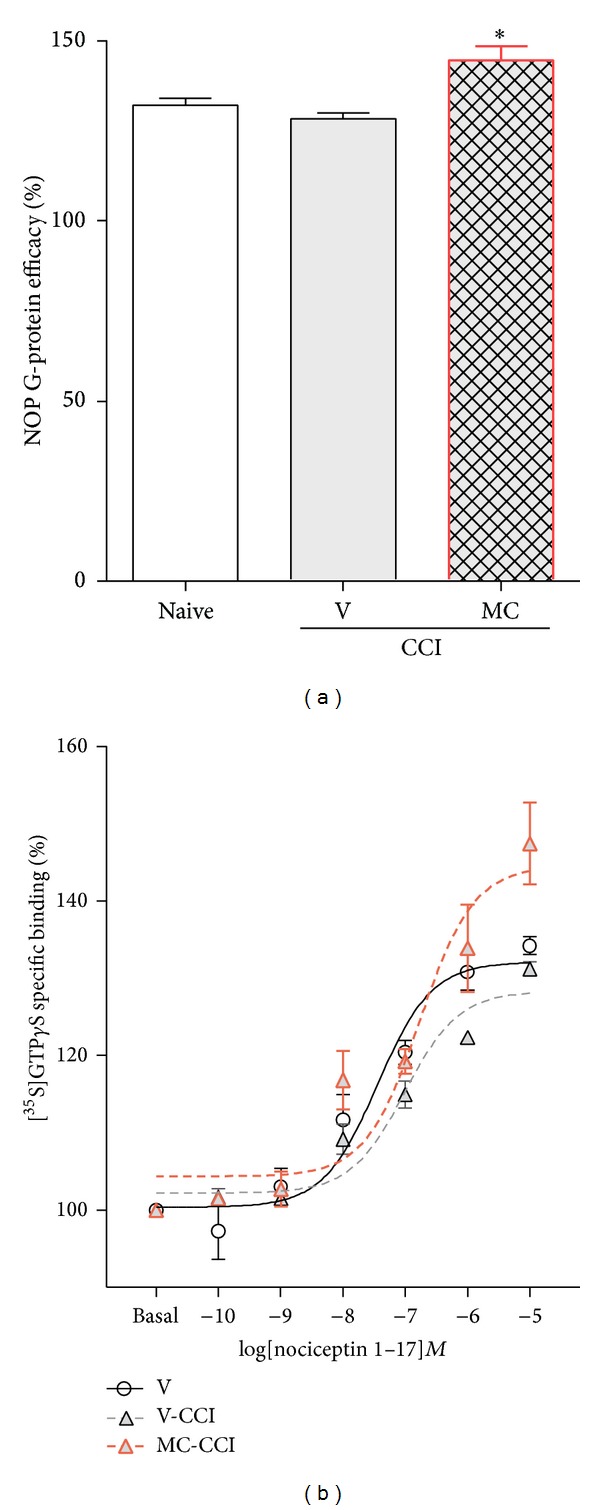
Repeated minocycline administration influenced NOP signaling. Chronic i.p. (30 mg/kg) minocycline treatment significantly increased the specific binding of the nucleotide analogue on NOP G-protein compared to vehicle-treated CCI-exposed rats. (a) The figure represents the calculated efficacy (or *E*_max⁡_) of the NOP-mediated G-protein during ligand stimulation. (b) The figure represents the specifically bound [^35^S]GTP*γ*S as a percentage in the presence of increasing concentrations (10^−10^−10^−5 ^M) of N/OFQ 1–17. Basal activity was settled as 100%. Points and columns represent mean ± SEM. for at least three experiments performed in triplicates. Intergroup differences were analyzed using ANOVA followed by Bonferroni's multiple comparison test. *P < 0.05 indicates significant differences compared with naïve rats. V: vehicle, MC: minocycline.

In the [^35^S]GTP*γ*S binding assay, during N/OFQ stimulation in the ipsilateral lumbar dorsal rat spinal cord CCI-exposed rats remained unaffected compared to the naive group. However, repeated minocycline treatment markedly increased the specific binding of the nucleotide analogue during NOP-mediated G-protein activation (Figure 3(b)). This resulted in a significant increase in the maximal activation (or efficacy, *E*_max⁡_) of NOP-mediated G-protein compared with the vehicle-treated CCI-exposed group (Figure 3(a)). The potency (pEC_50_) of the ligand remained unaltered (data not shown; the curves did not shift to either side to a significant degree) compared to naive either to vehicle treated CCI-exposed animals (Figure 3(b)).

### 3.4. Minocycline Influenced the Nociceptin/Orphanin FQ Peptide Receptor mRNA but Not Protein Levels in Rat Primary Microglial Cell Cultures

**Figure 4:**
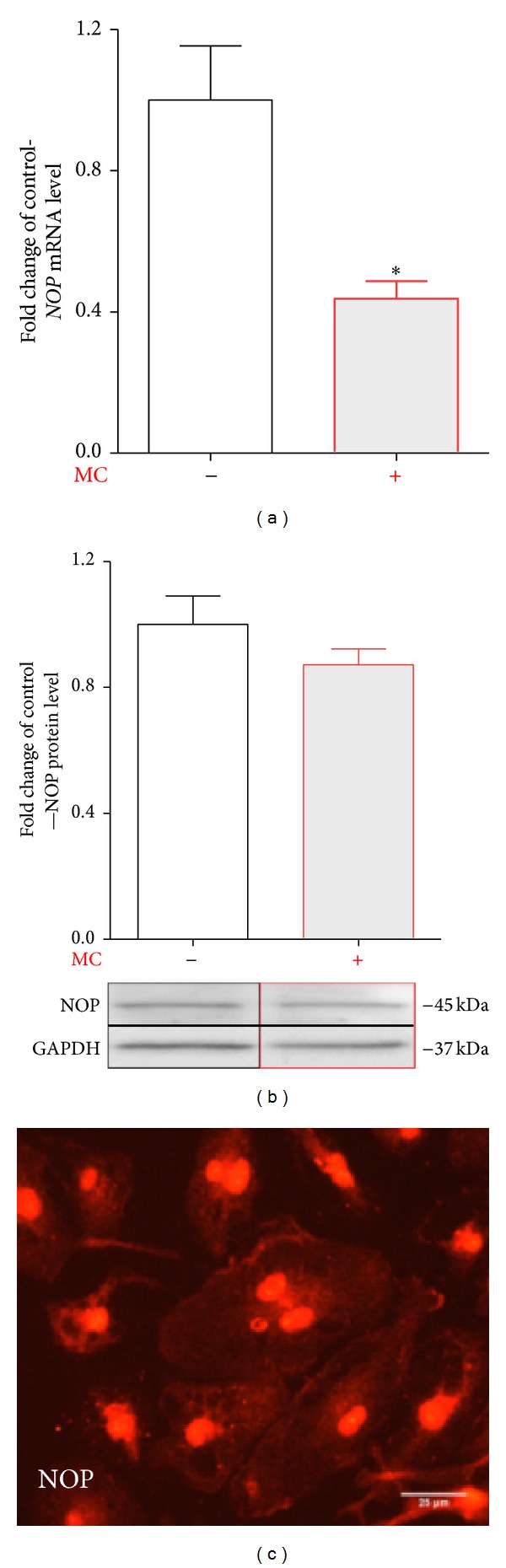
Minocycline diminished the mRNA but not protein NOP level in primary microglial cells. Primary microglial cell cultures were treated with minocycline [MC; 10 *μ*M] for 6 h for mRNA analysis (a) and 24 h for protein analysis (b). The qRT-PCR analysis shows that minocycline [10 *μ*M] downregulates* NOP* mRNA in primary microglial cell cultures (a). The Western blot analysis shows that minocycline did not change the protein level of NOP in primary microglia cultures (b). The qRT-PCR and Western blot data are presented as the mean ± SEM and represent the normalized averages derived from the analyses of four experiments. The intergroup differences were analyzed with a *t*-test; significant differences resulting from comparison with nonstimulated cells are indicated by *P < 0.05. The presence of NOP on microglial cells was confirmed by immunocytochemistry (c). The scale bar for all microphotographs is 25 *μ*m.

Rat primary microglial cell cultures were treated with minocycline (10 *μ*M) for 6 h and 24 h for mRNA and protein analysis, respectively. The qRT-PCR analysis shows that minocycline downregulates* NOP* mRNA in comparison with vehicle-treated cells (Figure 4(a)). Using Western blot analysis we have shown that minocycline did not have influence on the protein level of NOP after 24 h treatment in primary microglial cell cultures in comparison with vehicle-treated cells (Figure 4(b)). The presence of NOP in microglial cells was confirmed by immunocytochemistry (Figure 4(c)).

## 4. Discussion

Numerous pain studies are focused on the N/OFQ system because it is known that at the spinal cord level N/OFQ shows antinociceptive action through NOP, present in sensory neurons. NOP is considered as a novel potential target in the pain therapy. However, the role played by microglia in the functioning of this system has not been studied thus far. In the present study, we have shown for the first time the important influence of microglial activation on the effectiveness of the N/OFQ system. We demonstrated that repeated administration of minocycline potentiated the analgesic effects of N/OFQ in neuropathic rats and the effect seems to be additive. Chronic minocycline administration reduced the elevated spinal level of NOP protein of CCI-exposed rats, and it significantly increased NOP signaling possibly through the upregulation of NOP coupled G_i_ protein activation. Interestingly, in our* in vitro* studies we had shown that minocycline downregulates mRNA level for NOP in primary microglial cell cultures.

The N/OFQ analgesic activity is mediated selectively by NOP [3, 47, 48], which has been shown to act through the same intracellular pathway as classical opioid receptors [3, 7]. In healthy animals, N/OFQ administered i.t. has been generally found to produce antinociceptive responses that are similar to classical opioid receptor agonists without inducing signs of sedation or motor impairment [3, 10–14, 49, 50] which is why this system seems to be a promising target for chronic pain treatment. N/OFQ administered i.t. in neuropathic pain strongly alleviates both allodynia and hyperalgesia [4, 5, 48, 51, 52], and these results are in agreement with our behavioral results shown in the present paper. Additionally, electrophysiological studies have confirmed that the antinociceptive potency of spinally administered N/OFQ is maintained or even enhanced after nerve injury [14]. This is in contrast with classic opioid receptor agonists, such as morphine, which are less effective in neuropathic than in acute pain conditions, making the N/OFQ system a much more effective target in neuropathic pain treatment [53].

N/OFQ and its receptor are localized in nervous system regions that are associated with nociception [22–24, 54]. Using immunoreactivity-based approaches, NOP protein has been detected in the gray matter of the spinal cord, particularly in the superficial layer II of the lumbar dorsal horn [9, 27]. Therefore, for our biochemical analysis we used dorsal part of the lumbar spinal cord L4–L6. Luo et al. [55] have shown that in the dorsal horn N/OFQ suppresses excitatory, but not inhibitory (glycinergic or GABAergic), synaptic transmission to substantia gelatinosa neurons. It has been clearly documented that the induction of chronic pain states, especially neuropathy, is associated with a regulation of the N/OFQ system [51, 56, 57].

It is suggested that the development of neuropathic pain conditions is caused by abnormal glial cell activation, especially microglia [58–62]. However, there is a lack of information on whether NOP can be expressed and regulated on microglial cells in the spinal cord during neuropathy. It is well known that microglial cells in the spinal cord become activated in response to injury [17, 61, 63–65] and may modulate pain by producing pronociceptive substances, such as cytokines (IL-1beta, TNFalpha, IL-6, fractalkine, MIP-1alpha, MIP-1beta, and MCP-1), cytokine receptors (TNFRI, TNFRII, IL-1RI, and CX3CR1), cytotoxic compounds (iNOS, NO, ROS, and ATP), prostaglandins, and excitatory amino acids [62, 66–71]. In our previous studies, we have shown that minocycline diminished the development of neuropathic pain in CCI-exposed rats [41, 64]. In our experiments, we used minocycline, which besides some influence on neurons [72, 73], is considered as an inhibitor of the microglial activation [29–32], viability, and migration [33, 34] during neuropathic pain. In our previous experiments, we observed that minocycline can enhance the antiallodynic and antihyperalgesic effects of morphine, DAMGO, and U50,488H but not DPDPE, deltorphin II, or SNC80 [74]. In the present study, we demonstrated for the first time that in CCI-exposed rats the antiallodynic and antihyperalgesic effects of N/OFQ were significantly potentiated by minocycline, similar to opioid agonists.

Our biochemical study shows strong ipsilateral upregulation of protein for NOP parallel to microglial cell activation, while fewer changes are observed in minocycline-treated rats. In our earlier experiments using* in situ* hybridization, we have shown that the upregulation of* NOP* mRNA occurred only in the ventral but not the dorsal horn of ipsilateral lumbar part of the spinal cord, suggesting it is increased in motoneurons [6]. Similarly, Briscini et al. [51] showed the upregulation of* NOP* mRNA in the whole (dorsal and ventral) ipsilateral lumbar enlargement. In our present study, no changes in* NOP* mRNA level were observed in the spinal dorsal part at L4–L6 in either vehicle- or minocycline-treated rats compared to naive animals.

Our results suggest that the alterations in the spinal N/OFQ signaling by minocycline treatment change the effectiveness of coupled G_i_ proteins which in turn compensates for neuropathic pain. G-protein activation was monitored in functional [^35^S]GTP*γ*S binding assays using N/OFQ 1–17 to activate the receptor. The unaffected potency of N/OFQ shows that the binding site of the receptor is unaltered in CCI-exposed rats after vehicle or minocycline injection. We have shown that minocycline caused an increase in N/OFQ-stimulated NOP signaling and influenced the N/OFQ system functionality during neuropathic pain.

The downregulation of the NOP by minocycline is in parallel with a reduced level of IBA-1 protein, which suggests that microglia play an important role in this phenomenon. In 2010, Mika et al. [17] demonstrated by using immunohistochemistry that repeated minocycline treatment reversed the injury-induced activation of microglia/macrophages in the dorsal lumbar spinal cord. Our present data suggest that the changes in the NOP affect not only neurons but also results from glial cells activation, which is in agreement with previous reports [20, 21, 72]. Using primary microglial cell cultures, we have shown for the first time that minocycline downregulates the level of* NOP* mRNA in microglial cells. Our results may suggest that observed changes in NOP protein level after minocycline administration in a rat model of neuropathic pain can occur as result of the inhibition of microglial activation.

## 5. Summary

**Scheme 1:**
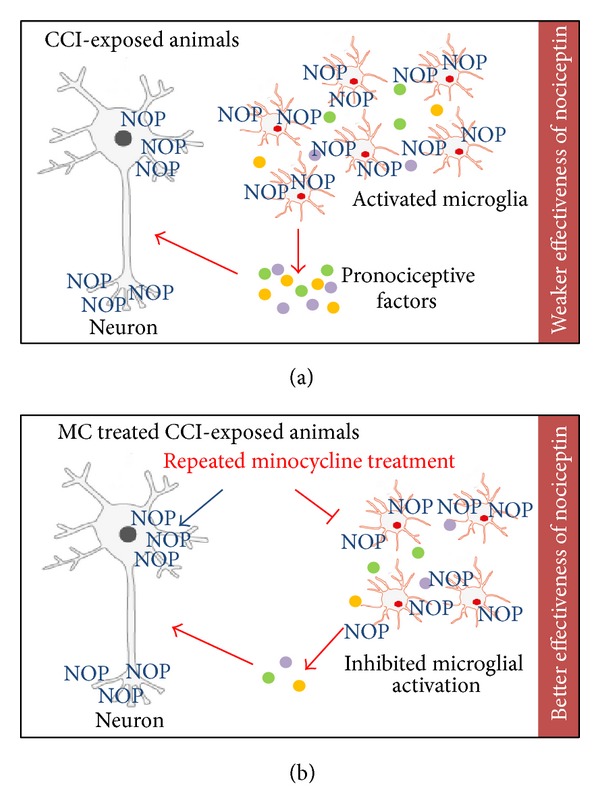
Based on our results, we hypothesize the possible influence of minocycline on the N/OFQ system during neuropathic pain. Activated spinal microglia are key factors in the development of neuropathic pain by producing pronociceptive substances [65] and they play a role in the efficacy of analgesics (a). Minocycline (MC) potentiated the effects of N/OFQ through the downregulation of microglial activation, which leads to decrease of the microglial pool of NOP at the spinal cord level. This action of minocycline leads to increasing the analgesic effects of N/OFQ through neuronal receptors (b).

Minocycline potentiates the effects of N/OFQ through the downregulation of microglial activation and also by decreasing the microglial pool of NOP. Thus, it increases the analgesic action of N/OFQ through neuronal receptors, and it also potentiates the receptor-ligand signaling through the upregulation of G-protein activation. The results of the present study provide evidence that minocycline not only diminishes neuropathic pain-related behavior, but also enhances the effectiveness of N/OFQ by modulation of NOP expression and activity (Scheme 1). Our findings suggest that activated spinal microglial cells, which are the key factors in the development of neuropathic pain, play an important role in the function of the N/OFQ system. Therefore, specific microglial modulators, such as minocycline, combined with N/OFQ may be an interesting target to develop new therapy that would be effective against neuropathic pain.
